# Biomechanical comparison of screw-based zoning of PHILOS and Fx proximal humerus plates

**DOI:** 10.1186/s12891-018-2185-5

**Published:** 2018-07-25

**Authors:** Ali Jabran, Chris Peach, Zhenmin Zou, Lei Ren

**Affiliations:** 1 School of Mechanical, Aerospace and Civil Engineering, University of Manchester, Sackville Street, Manchester, M13 9PL UK; 2 Department of Shoulder and Elbow Surgery, University Hospital of South Manchester, Southmoor Road, Wythenshawe, Manchester, M23 9LT UK

**Keywords:** Proximal humerus fractures, Biomechanical testing, Locking plate, Blade plate

## Abstract

**Background:**

Treatment of proximal humerus fractures with locking plates is associated with complications. We aimed to compare the biomechanical effects of removing screws and blade of a fixed angle locking plate and hybrid blade plate, on a two-part fracture model.

**Methods:**

Forty-five synthetic humeri were divided into nine groups where four were implanted with a hybrid blade plate and the remaining with locking plate, to treat a two-part surgical neck fracture. Plates’ head screws and blades were divided into zones based on their distance from fracture site. Two groups acted as a control for each plate and the remaining seven had either a vacant zone or blade swapped with screws. For elastic cantilever bending, humeral head was fixed and the shaft was displaced 5 mm in extension, flexion, valgus and varus direction. Specimens were further loaded in varus direction to investigate their plastic behaviour.

**Results:**

In both plates, removal of inferomedial screws or blade led to a significantly larger drop in varus construct stiffness than other zones. In blade plate, insertion of screws in place of blade significantly increased the mean extension, flexion valgus and varus bending stiffness (24.458%/16.623%/19.493%/14.137%). In locking plate, removal of screw zones proximal to the inferomedial screws reduced extension and flexion bending stiffness by 26–33%.

**Conclusions:**

Although medial support improved varus stability, two inferomedial screws were more effective than blade. Proximal screws are important for extension and flexion. Mechanical consequences of screw removal should be considered when deciding the number and choice of screws and blade in clinic.

**Electronic supplementary material:**

The online version of this article (10.1186/s12891-018-2185-5) contains supplementary material, which is available to authorized users.

## Background

Proximal humerus fractures are relatively common injuries, accounting for 5–8% of all fractures [1, 2]. They are more prevalent in the over-60 female population group [3]. Incidence of these fractures, especially in the elderly patients after low energy falls, is increasing due to the growing elderly population with osteoporosis [4]. Younger patients, however, generally sustain them by high energy traumas [5].

Approximately 80% of proximal humerus fractures are stable with low displacement of fracture fragments, so their conservative management has proven to be successful with high patient satisfaction [6]. The remaining 15–20% fracture cases are characterised by instability and significant displacements so surgical intervention is required to restore stability, improve chances of bone healing and allow early rehabilitation. Although there are many implants available for treatment of these fractures, the optimum method of fracture fixation is unclear [7].

Since the development of locking technology several decades ago, biomechanical studies have shown advantages of locking plates over conventional non-locking and blade plates [8–10]. Clinical studies, on the other hand, reveal high complication rates with their use, often necessitating revision surgery [11–13]. Common complications include varus deformity, screw cut-out and screw penetration through the humeral head and into the glenohumeral joint [14–16]. In light of this, Gardner et al. suggested the importance of medial support for maintaining fracture reduction [17].

Although positive outcomes have been achieved with the medial insertion of autologous bone grafts, fibular allograft, calcium phosphate bone cement and inferomedial screws in plates, there is no golden standard for the medial support reconstruction [13, 18–21]. Despite this, inferomedial screws have become a common feature of recent locking plate design. One such plate is the PHILOS (Proximal Humerus Internal Locked System) plate (Synthes, Paoli, Pennsylvania, USA) that allows fixed angle insertion of two locking inferomedial screws. With a similar design philosophy, the Equinoxe Fx plate (Exactech, Gainsville, FL) is a hybrid fixed angle blade plate that provides the option of implanting inferomedial locking screws or a blade. The rationale for a hybrid system that allows both blade and screws in a single plate is to reap the most from the mechanical benefits of locking screw technology and the fracture buttressing provided by the increased surface area of a blade. There is a scarcity of literature on the mechanical contribution of the blade- or screw-based inferomedial support as compared to screws of other parts of the proximal humerus plate.

The aim of this study was two-fold. Firstly, we aimed to determine the effect of lack of medial support, both in form of inferomedial locking screws and blade, on the extension, flexion, valgus and varus bending stiffness of humeri treated with either PHILOS or Fx plate. Secondly, we aimed to investigate the effect of removal of other humeral head screws on the extension, flexion, valgus and varus bending stiffness of humeri treated with either PHILOS or Fx plate.

## Methods

Forty-five left synthetic humeri (model 1028; Pacific Research Laboratories, Vashon, WA, USA) were obtained and divided into nine groups, each containing five specimens. Five groups were implanted with 90 mm stainless steel PHILOS locking plate and the remaining four groups were implanted with 80 mm stainless steel Fx fixed-angle locking blade plate.

**Fig. 1:**
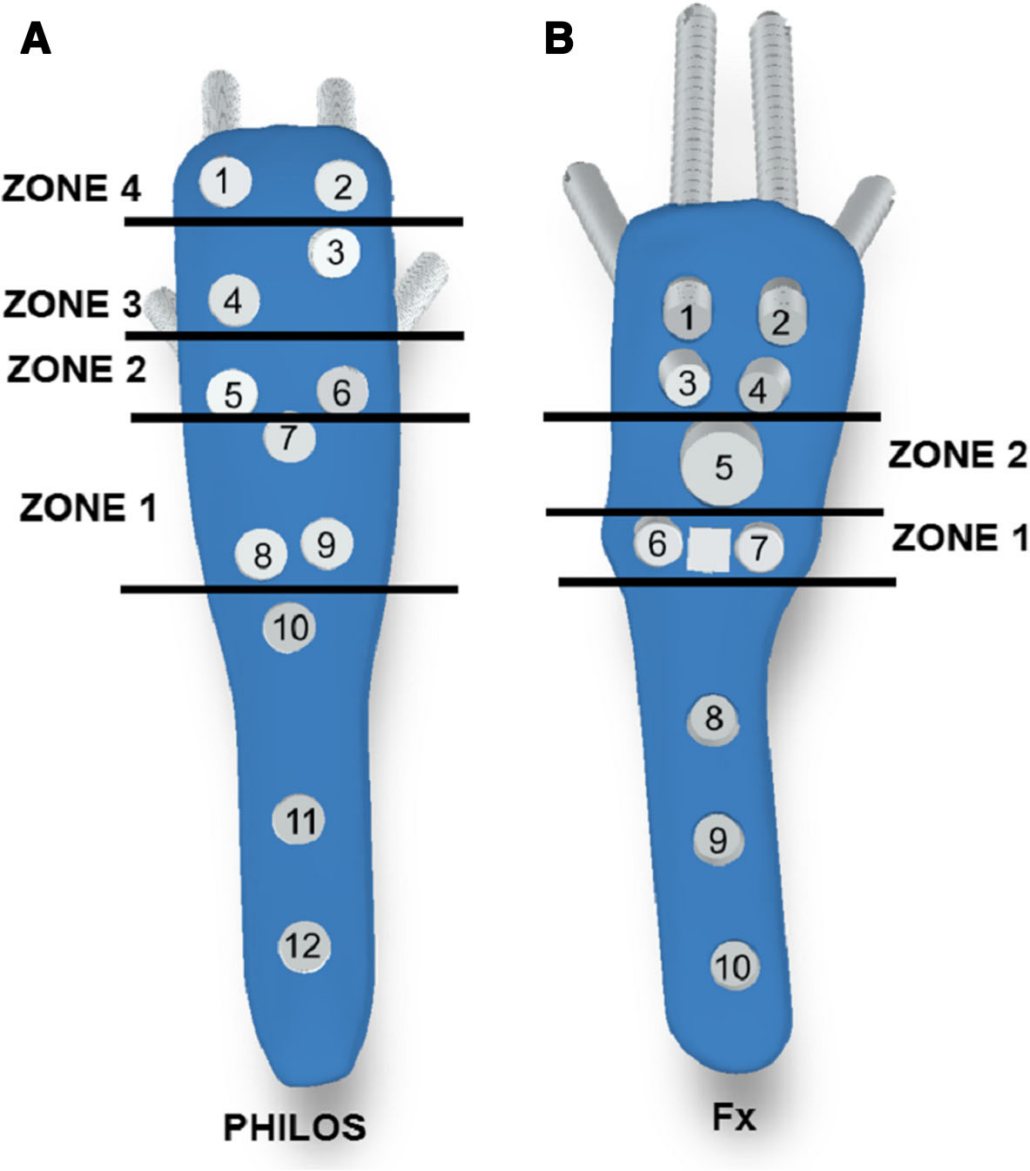
Numbering and zoning of screws and blade holes on PHILOS plate (**a**) and Fx plate (**b**) based on their proximity to fracture gap]

Screws and blades of both PHILOS and Fx plate were numbered and then categorised into several zones based on their positions on the plates (Fig. 1). Of the five groups implanted with PHILOS plate, four had either zone 1, 2, 3 or 4 screws missing (P1-P4) while the fifth group (P0) acted as the control configuration group as it had all the zones filled. Similarly, two of the four groups implanted with Fx plate had either zone 1 (F1) or zone 2 (F2) missing while the specimens from the third group had the blade swapped with two inferomedial locking screws (F3). The fourth Fx group had all the zones filled and acted as a control configuration group (F0). Details of the screw and blade choice for each plate configuration are tabulated in Table 1. Length of screws and blades were selected so that their tips abutted the subchondral bone, with the aim of achieving maximum screw purchase. This was determined in trial studies using the depth gauges provided by the manufacturers.

**Table 1:** Length (mm) and descriptions of the screws and blades for the PHILOS plates and Fx plate configuration groups. Emboldened cells correspond to where combination differs from the control. Types of screws, cancellous, cortical locking and cortical compression, are denoted by CA, CO-L and CO-C, respectively

Configuration Group	Screw Number											
	1	2	3	4	5	6	7	8	9	10	11	12
P0 (Control)	40	40	45	50	42	42	None	50	50	32	30	30
P1 (No Zone 1)	40	40	45	50	42	42	None	None	None	32	30	30
P2 (No Zone 2)	40	40	45	50	None	None	None	50	50	32	30	30
P3 (No Zone 3)	40	40	None	None	42	42	None	50	50	32	30	30
P4 (No Zone 4)	None	None	45	50	42	42	None	50	50	32	30	30
F0 (Control)	29, CA	29, CA	44, CA	44, CA	50	45, Blade		26, CO-L	32, CO-C	26, CO-L	N/A	N/A
F1 (No Zone 1)	29, CA	29, CA	44, CA	44, CA	50	None		26, CO-L	32, CO-C	26, CO-L	N/A	N/A
F2 (No Zone 2)	29, CA	29, CA	44, CA	44, CA	None	45, Blade		26, CO-L	32, CO-C	26, CO-L	N/A	N/A
F3 (Swap Blade with Screws)	29, CA	29, CA	44, CA	44, CA	50	44, CA	44, CA	26, CO-L	32, CO-C	26, CO-L	N/A	N/A

**Fig. 2:**
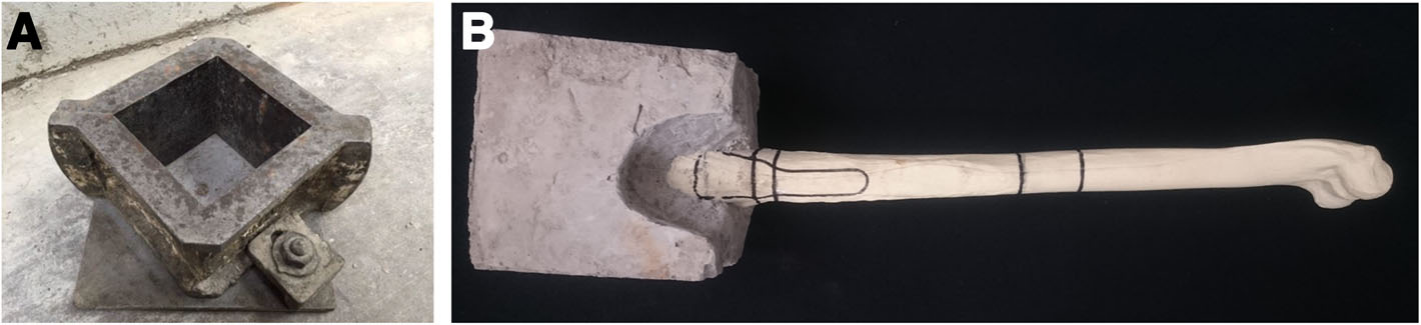
Specimen preparation: Cubic mould (**a**) was filled with cement mix and humerus. After the block dried, humerus (**b**) was marked with positions of plate and cuts

All specimens were potted in cement blocks to allow easy clamping of the humeral heads to the testing machine during the biomechanical tests (Fig. 2). The blocks were cubic so that by setting each of their four faces parallel to either the sagittal or frontal planes, loads could be applied along the anatomically accurate directions. With the shaft clamped vertically, humeral head was placed into a 100 cm^3^ cubic mould. A cement mixture, consisting of standard, general purpose (Portland limestone) cement, rapid mix cement and water, was prepared by a ratio of 4:1:2.5 by volume. For homogeneity, the three were mixed using an electric mixer and then poured into the mould. The resulting mixture submerged the humeral head and was left for 48 h in the mould to dry.

Once removed from the mould, specimens were sawed at shaft to a length of 210 mm from humeral head apex. A 10 mm half-cut was created at the surgical neck. To simplify the process of fixating the plate onto humerus, plates were attached to the bone prior to the removal of the fracture piece. A half-cut was carried out to ease the removal of the fracture piece later and also to prevent damage caused to the plate.

The superior ends of the PHILOS and Fx plate were positioned 30 and 12 mm distal to the superior greater tuberosity. Once the plates were snug to the bone, screws holes were drilled and screws were implanted, starting from the shaft screws. All specimens were implanted according to the manufacturers’ guidelines. The plates had similar procedures for specimen preparation, with small differences because of design features such as the blade insertion required in Fx plate. For the Fx plate, blades were inserted using a blade osteotome and held in position by shoulders grub screws. After implantation, the removal of the 10-mm block of bone was achieved by cutting through the other side of the bone to meet the previous two cuts and simulate a two-part fracture (corresponding to Neer classification). The block of bone was gently knocked out to prevent damage to the plate or any of the screws.

**Fig. 3:**
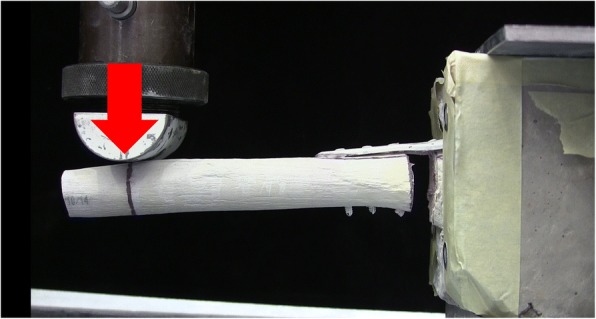
Mechanical testing set-up used to apply varus cantilever displacement (red arrow) and determine bending loads and stiffness. Load was applied to the humeral shaft in a cantilever fashion while humeral head was potted inside the cement block

All forty-five specimens were subjected to both elastic (varus, valgus, extension and flexion bending five times) and plastic testing (varus bending once). For elastic testing, specimens were placed in an Instron 4500 universal materials testing machine (Instron, Canton, MA, USA) such that the humeral shaft was in a horizontal orientation. The specimens were then clamped rigidly at their proximal end by a custom fixture. The testing machine was installed with a semi-circular prismatic shaft holder. This way, crosshead was in contact with the humeral shaft at a distance of 30 mm from the specimen’s distal end (Fig. 3). Five-millimetre displacement was applied at 1 mm/s along the frontal plane to achieve varus cantilever bending and the crosshead was then retracted back to its original position. This displacement was applied five times, after which the specimen was offloaded and repositioned so that it could be displaced along the frontal plane but in the opposite direction to induce valgus bending. In a similar manner, displacement was applied along the sagittal plane for extension and flexion bending.

Based on the load and displacement data recorded from the testing machine, peak load at 5 mm (F_5_) and elastic stiffness (K) were determined for each direction.

Subsequently, plastic testing was conducted on all specimens to investigate their varus stability under large displacements. For this, the position of the constructs was similar to that for the varus elastic tests. Specimens were displaced at a rate of 0.05 mm/s until a 15-mm displacement was achieved. After an eight-minute intermission, the displacement was resumed at the same rate until a 30-mm displacement was obtained. Based on the trial studies, 30 mm was found to be large enough to ensure that all the specimens were in the plastic region of their load-displacement curve. For these tests, load at 30 mm (F_30_) and those before and after the 15-mm intermission (F_15a_ and F_15b_) were determined using the load-displacement data.

The statistical analyses of the experimental data were conducted using the SPSS 22.0 software (IBM, NY, USA). The effects on specimen groups’ stiffness and load values were analysed by using a linear mixed model approach by taking intra- and inter-subject variability into account.

The fixed effect in the analysis was the configuration group while the specimens and their trials were set as the random effects. Dependent variables in the elastic test data were K and F_5_ but in plastic test data, they were F_15a_, F_15b_ and F_30_. The pair-wise difference was tested using Fisher’s least significant difference (LSD) multiple comparison based on the least-squared means.

## Results

### Elastic testing results

**Fig. 4:**
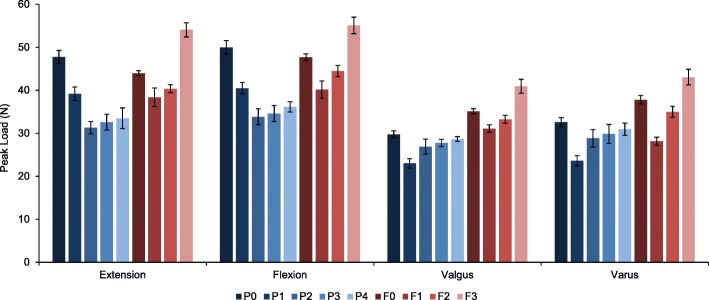
Mean peak load (F5) for PHILOS and Fx plate configuration groups during elastic loading of 5 mm cantilever displacement in extension, flexion, valgus and varus directions

**Fig. 5:**
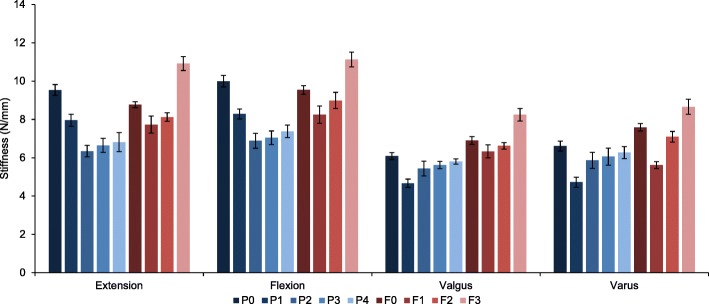
Mean stiffness (K) for PHILOS and Fx plate configuration groups during elastic loading of 5 mm cantilever displacement in extension, flexion, valgus and varus directions

For both plates, the trends in the mean peak loads among configuration groups were similar to those obtained for their mean stiffness (Figs. 4 & 5). No implant failure or screw pull-out was observed for any specimen during all tests. Out of all zones tested for PHILOS plate, removal of zone 1 screws in P1 had the greatest effect on valgus and varus stability, leading to 23 and 28% drop in mean stiffness (mean stiffness: 4.671/4.726 N/mm) when compared to the control group P0 (Table 2). In the order of decreasing effect on varus stiffness with their removal, zone 1 screws were followed by screws of zone 2 (5.867 N/mm), zone 3 (6.059 N/mm) and zone 4 (6.268 N/mm). This not only highlighted the importance of inferomedial screws for varus stability but also the likely link between the screws’ position and varus stiffness. Removal of zone 1 screws had least impact on mean stiffness values in extension and flexion (7.956/6.349 N/mm). For loading along these two directions, removal of zone 2 led to the largest drop (33 and 31%) in mean stiffness (6.349/6.887), followed by the removal of screws of zone 3 (6.644/7.045 N/mm), zone 4 (6.871/7.377 N/mm) and zone 1 (7.956/8.284 N/mm).

**Table 2:** Mean stiffness (K) and load values (F) for all PHILOS plate configuration groups, along extension, flexion, valgus and varus, with their respective standard deviations (S.D.). K and F5 denote stiffness and peak load values obtained during elastic tests while F15a and F15b are loads at 15 mm before and after eight-minute intermission and F30 is the load at 30 mm during plastic tests

	P0 (SD)	P1 (SD)	P2 (SD)	P3 (SD)	P4 (SD)
Extension					
K (N/mm)	9.533 (0.286)	7.956 (0.314)	6.349 (0.299)	6.644 (0.365)	6.819 (0.495)
F_5_ (N)	47.749 (1.510)	39.191 (1.580)	31.295 (1.425)	32.597 (1.842)	33.501 (2.415)
Flexion					
K (N/mm)	9.997 (0.298)	8.284 (0.257)	6.887 (0.391)	7.045 (0.357)	7.377 (0.331)
F_5_ (N)	49.981 (1.569)	40.475 (1.336)	33.846 (1.876)	34.601 (1.850)	36.152 (1.196)
Valgus					
K (N/mm)	6.091 (0.181)	4.671 (0.2150)	5.439 (0.386)	5.623 (0.189)	5.804 (0.140)
F_5_ (N)	29.746 (0.815)	23.041 (1.065)	26.907 (1.769)	27.765 (0.852)	28.659 (0.537)
Varus					
K (N/mm)	6.609 (0.256)	4.726 (0.259)	5.867 (0.417)	6.059 (0.443)	6.268 (0.317)
F_5_ (N)	32.561 (1.075)	23.601 (1.183)	28.826 (2.041)	29.862 (2.205)	30.951 (1.436)
F_15a_ (N)	75.590 (3.049)	46.636 (1.843)	53.759 (1.513)	62.235 (1.941)	65.012 (2.632)
F_15b_ (N)	71.558 (3.303)	42.376 (2.141)	50.199 (2.118)	58.432 (1.878)	62.693 (3.592)
F_30_ (N)	115.531 (6.336)	70.077 (3.446)	81.238 (3.127)	95.103 (2.901)	103.216 (5.422)

In extension, flexion, valgus and varus testing of Fx plate, mean stiffness with the swapping of the blade with inferomedial screws in F3 (10.915/11.127/8.245/8.663 N/mm) was higher than the control group F0 (Table 3), followed by the removal of 6.5 mm screws in F2 (8.122/8.990/6.623/7.094 N/mm) and blade in F1 (7.734/8.248/6.332/5.619 N/mm). Like the removal of zone 1 screws in PHILOS plate, removing blade in Fx plate (F1) led to a larger decrease in valgus and varus stiffness (8 and 26%) than the removal of 6.5 mm screw (F2) when compared to control group F0. However, unlike the PHILOS plate configuration groups where zone 2 screws had a greater effect on extension and flexion than screws from zone 1, 6.5 mm screw in the Fx plate had less impact on extension and flexion stiffness than the removal of the blade (8.1218/8.990 vs 7.734/8.248 N/mm). Swapping the blade with screws led to a statistically significant increase in extension, flexion, valgus and varus stiffness than the control group (10.915/11.127/8.245/8.663 vs 8.770/9.541/6.900/7.590 N/mm).

**Table 3:** Mean stiffness (K) and load values (F) for all Fx plate configuration groups, along extension, flexion, valgus and varus, with their respective standard deviations (S.D.). K and F5 denote stiffness and peak load values obtained during elastic tests while F15a and F15b are loads at 15 mm before and after eight-minute intermission and F30 is the load at 30 mm during plastic tests

	F0 (SD)	F1 (SD)	F2 (SD)	F3 (SD)
Extension				
K (N/mm)	8.770 (0.156)	7.734 (0.445)	8.122 (0.220)	10.915 (0.362)
F_5_ (N)	43.979 (0.596)	38.394 (2.151)	40.357 (0.927)	54.071 (1.651)
Flexion				
K (N/mm)	9.541 (0.221)	8.248 (0.454)	8.990 (0.424)	11.127 (0.385)
F_5_ (N)	47.711 (0.775)	40.160 (2.033)	44.479 (1.321)	55.095 (1.922)
Valgus				
K (N/mm)	6.900 (0.200)	6.332 (0.339)	6.623 (0.170)	8.245 (0.324)
F_5_ (N)	35.131 (0.617)	31.096 (0.855)	33.260 (0.919)	40.946 (1.600)
Varus				
K (N/mm)	7.590 (0.196)	5.619 (0.180)	7.094 (0.280)	8.663 (0.391)
F_5_ (N)	37.792 (0.990)	28.151 (0.946)	35.001 (1.277)	43.059 (1.833)
F_15a_ (N)	84.470 (1.547)	81.472 (2.665)	73.545 (1.303)	90.735 (2.439)
F_15b_ (N)	79.304 (2.507)	78.650 (2.327)	70.296 (1.547)	87.577 (2.294)
F_30_ (N)	134.391 (3.574)	128.636 (2.339)	123.032 (6.161)	141.294 (3.487)

Results from the statistical analysis of the PHILOS configuration groups showed that there were statistically significant differences (*P* values less than 0.05) between stiffness and load values of all configuration group pairs, except two cases (Additional file 1 and Additional file 2). These were P3 and P4 in extension, and P2 and P3 in flexion. As for the pairwise comparison of the Fx plate configuration groups, there were statistically significant differences between peak loads and stiffness values of all configuration pairs (Additional file 3).

### Plastic testing results

**Fig. 6:**
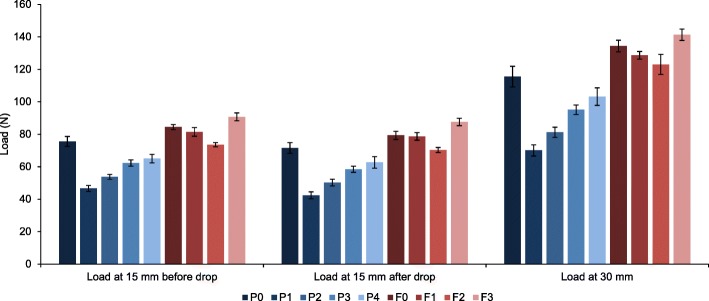
Mean peak loads (F) for PHILOS and Fx plate configuration groups during plastic loading at 15 mm displacement before (F_15a_) and after (F_15b_) eight-minute intermission and at 30 mm displacement (F_30_)

For both plates, the load trends recorded for the plastic tests among the configuration groups were similar to those recorded for elastic varus tests (Fig. 6). This showed that the conclusions drawn from varus elastic test tests remained relevant even when specimens were subjected to larger displacements. There were statistically significant differences for all configuration group pairs (Additional file 4 and Additional file 5) with only one exception, which were the F_15b_ values for the F0 and F2 configuration group pair.

## Discussion

The decision of how many and which screws to implant or leave out is a crucial one that is frequently made by clinicians. We believe that this decision is based on many factors including the screw’s location, orientation and geometry. There is very little information in the literature on the optimal number of screws for a given fracture, with two studies recommending the insertion of at least five screws in the humeral head including at least one inferomedial screw [22, 23].

Although biomechanical models do exist for guidance on optimal selection of these factors, they are often based on simple fracture types and loadings. Thus, conclusions drawn from them are not fully applicable and ought to be taken with caution especially with regards to treatment of complex fractures like proximal humerus fractures. Nevertheless, they are beneficial for understanding the results obtained in this study.

### Effects of screw location

A commonly studied analytical model in the literature is that of the bending behaviour of two-part fracture in a cylindrical bone specimen treated with a plate [24–26]. Regarding this, Smith et al. defined the plate working length as the distance between the closest two screws on either side of the fracture gap [25]. We kept the number of shaft screws constant so the plate working length could only be changed by the filling or emptying of zones neighbouring the fracture gap.

If this length is kept too short, the overall construct stiffness is increased but so is the risk of high-stress concentration, straining and eventual failure of the plate at the section around the fracture gap [27]. These negative effects are more profound in the osteopenic bone where the bone-metal interface is weak, leading to screws cut-out, the second fracture at plate end and reduction in the micromotion needed for postoperative callus formation [28–30].

In response to this, the concept of ‘semi-rigid’ plates arose, where some flexibility and movement is permissible at the fracture site in order to absorb the load energy and reduce the strain at the bone-metal interface [31, 32]. However, care must be taken during fracture healing to prevent excessive movements keep the fracture fragments undisturbed and intact enough to avoid early failure [28].

This flexibility could be achieved by keeping the screws holes near fracture site empty to increase the working length and keep stress and strain low and well distributed. This comes at the expense of a reduction in overall construct stiffness and stability. For example, removing zone 1 screws in PHILOS plate increased the moment arm which reduced the cantilever load required to produce the same moment, leading to reduced stiffness. Mechanically, this is similar to the case of a cantilever beam with a concentrated load at the free end where maximum deflection of the beam has a cubic relationship with its arm length.

In our study, P2, P3 and P4 configuration groups had zone 1 screws in place, so the working length was fixed. This would imply that for a given load direction, the bending stiffness of these configurations would be same. Instead, the effect of screw removal on overall construct stiffness decreased from zone 2 to 3 and eventually zone 4. This decline at each proximal progression is possibly because leaving out zone 2 screws created a gap in the plate. Further, this omission of screws near the fracture site affected the plate stability and construct stiffness. For P3 constructs, zone 1 and 2 screws were already present between zone 1 and the fracture gap. So, theoretically, they would exhibit higher stiffness than constructs with P2 configuration. Similarly, P4 constructs had zone 1, 2 and 3 screws in place and were stiffer than P3 constructs. Therefore, as the gap in the plate was made more proximal from the fracture gap, its influence over fracture gap stability and the construct stiffness diminished.

Based on these principles acquired from the simple analytical model, one could argue that it is the proximity of a zone to the fracture gap that dictates its importance in construct stability, as demonstrated by Stoffel et al. [27]. In varus and valgus bending, this was indeed true as the zones could be listed in the following order of reducing the effect on construct stiffness: zone 1, 2, 3 and 4.

### Effects of screw orientation

In extension and flexion bending, zone 2 screws had the largest effect on construct stiffness, followed by screws of zone 3, 4 and 1. This implies that for these two directions, construct stability is controlled by other factors in addition to screw placement. The inadequacy of the aforementioned model to predict this behaviour is possibly because it does not account for complex humerus geometry and the fact that PHILOS plate allows implantation of several, multidirectional screws in each zone.

The angle between the zone’s screw pairs and the plate midline seems to affect the extension and flexion bending stiffness. The near-parallel screw pairs had a lower impact on construct stability than diverging and converging screws. Zone 2 screws of PHILOS plate diverged by a relatively large angle while zone 3 screws had convergent trajectories. Zone 4 and 1 screws, on the other hand, were almost parallel to each other.

We believe that there are two main motives behind a screw orientation choice. The first is purely mechanical: to achieve enhanced stability along the load direction of interest. We observed this for extension and flexion where orienting the screws in these directions yielded higher stiffness. The second motive is biological: to connect regions of the humeral head with low bone quality to those of high bone quality such as the medial region [33, 34].

Medial support in plates, both in form of screws and blade, targets the inferomedial region. Importance of these screws for minimising humeral head collapse in varus bending is well known, but less so on that of the blade [22, 35, 36]. In a similar fashion to PHILOS plate’s zone 1 screws, the importance of the blade for varus stability in the Fx plate was manifested when it was removed, causing a 26% drop in varus mean stiffness, more than any of the other three directions. However, since the bone specimen we used was made out polyurethane foam with relatively uniform density, the superior performance of medial support was not attributable to the better bone mineral density. Instead, we believe that the main factor was the proximity of the screws and blade to the fracture site. The use of synthetic bone was both an advantage and disadvantage of our study. It was an advantage in the sense that it highlighted that medial support is vital for varus stability, irrespective of local variations in bone mineral density. At the same time, it was a simplification of the in vivo scenario and thus demands future testing on cadaveric specimens.

### Effects of screw geometry

Despite the significant importance of medial support over other zones, particularly in varus, a large difference was found between the medial support provided by zone 1 screws than that by the blade. Fx plate control group (F0) had superior varus and valgus stiffness values than PHILOS plate control group (P0) suggesting the advantage of using a blade. Swapping the Fx plate’s blade with locking screws increased construct stiffness even further, not only in varus but also in the other three directions. This could be an issue of geometry and consequently the indirect locking mechanism of the blade. Unlike most conventional blade plates, Fx plate and its blade exist as two separate parts. In order to connect the two, the blade is placed into its slot on the plate and two grub screws are inserted on the plate, just above its blade’s shoulders. This way, it is held in its place by the interference between its shoulders and the grub screws connected to the plate. There is also a geometrical mismatch between the rectangular cross-section of blade’s shoulders and the circular screw holes. Loosening of the grub screws would allow the blade to toggle and slide out. On the contrary, the two inferomedial screws do not rely on any grub screws and directly lock to the plate. In this way, they have longer effective working length and thus less toggling when subjected to bending. This issue of getting the blade, which has non-locking shoulders, to fix more rigidly to the plate should be addressed in future plate design.

The Fx plate’s large central hole in zone 2 permits the insertion of a 6.5 mm locking screw. Our results showed that when compared to the blade and the inferomedial screws, this screw had less impact on construct stiffness in all four bending directions. In light of our understanding of the effect of screw’s proximity to fracture gap in PHILOS plate, this is understandable since the 6.5 mm screw is more distant from the fracture than zone 1. We hypothesise that if two small screws both offset from plate midline were used instead of one large 6.5 mm screw, the bending stiffness, particularly in extension and flexion cantilever bending would be improved since the offset would increase the second moment of area. One advantage of the current large central hole is that it allows deployment of bone-void filler. Biomechanical benefits of cement augmentation have been demonstrated in several studies [37–42]. It may be useful, in future studies, to take the most stable configuration group from this study (F3), use bone cement in place of the 6.5 mm screw and investigate whether its augmentation further improves stability.

One way to modify the geometry of a screw or a blade is by changing its length. We idealised the screw purchase by keeping the screws long enough to achieve subchondral bone abutment. Due to the irregular geometry of the humeral head, screws were of varying lengths. Their possible influence on the mechanical performance of different zones can, therefore, not be ignored. Glenohumeral perforation of the screws is one of the leading complications associated with angle stable plates [14, 15, 43]. Using screws of shorter length may prevent screw perforation but can also lead to poor bone anchorage since the density of the cancellous bone in the subchondral region is relatively high [44]. As a consequence of this, the construct can lose its stability and collapse in varus.

## Conclusions

Addition of medial support, both in form of screws and blade, improved mean varus bending stiffness of PHILOS and Fx plate specimens and we attribute this to their proximity to fracture gap. However, further studies on cadaveric specimens are needed to account for the effects of bone density on screw anchorage. Results showed that the type of medial support matters. In the Fx plate, the medial support provided by inferomedial screws exhibited significantly superior extension, flexion, valgus and varus bending than that by the blade.

Screw pairs placed proximal to the fracture gap played a significant role on extension and flexion bending stiffness, possibly owing to their non-parallel orientation. Hence, in appreciation of the complexity of in vivo loading of the humerus, we conclude that for general stability all four zones are critical as they have a synergistic relationship and clinical decisions ought to be made depending on the nature of the fracture being treated. The relatively low effect of the use of large 6.5 mm screw in Fx plate as compared to that of zone 2 screws on PHILOS plate demands a further mechanical investigation.

It is hoped that findings of the present study will provide valuable information to the clinicians with the decision making involved in selecting the optimal number and configuration for a given fracture case. It is also hoped that the design choices discussed in this study, especially with regards to the location, orientation and geometry of the screws and the locking mechanism of blades, will assist the design of future proximal humerus plates with enhanced mechanical and clinical performance.

## Additional files

Additional file 1: Raw experimental data for calculation of mean stiffness and peak loads and for performing statistical analysis. (XLSX 73 kb)

Additional file 2: P values for elastic stiffness and peak load values of PHILOS plate configuration groups, obtained from their pairwise comparison statistical analysis (DOCX 17 kb)

Additional file 3: P values for elastic stiffness and peak load values of Fx plate configuration groups, obtained from their pairwise comparison statistical analysis. (DOCX 16 kb)

Additional file 4: Results of pairwise comparison statistical analysis: *P* values for plastic load at 15 mm displacement before (F_15a_) and after (F_15b_) eight-minute intermission and at 30 mm displacement (F_30_), for PHILOS plate configuration groups. (DOCX 15 kb)

Additional file 5: Results of pairwise comparison statistical analysis: *P* values for plastic load at 15 mm displacement before (F_15a_) and after (F_15b_) eight-minute intermission and at 30 mm displacement (F_30_), for Fx plate configuration groups. (DOCX 15 kb)
