# Supplementary material for: Biomechanical comparison of screw-based zoning of PHILOS and Fx proximal humerus plates
Source: BMC Musculoskelet Disord. 2018 Jul 25;19:253. doi: 10.1186/s12891-018-2185-5 (PMC6060456; doi:10.1186/s12891-018-2185-5)
Supplement: Supplementary file 2 — P values for elastic stiffness and peak load values of PHILOS plate configuration groups, obtained from their pairwise comparison statistical analysis (DOCX 17 kb) [file 12891_2018_2185_MOESM2_ESM.docx]

|  | Peak Load (F_5_) | | | | Stiffness (K) | | | |
| --- | --- | --- | --- | --- | --- | --- | --- | --- |
| Direction/Zone | P1 | P2 | P3 | P4 | P1 | P2 | P3 | P4 |
| Extension |  |  |  |  |  |  |  |  |
| P0 | <0.001 | <0.001 | <0.001 | <0.001 | <0.001 | <0.001 | <0.001 | <0.001 |
| P1 |  | <0.001 | <0.001 | <0.001 |  | <0.001 | <0.001 | <0.001 |
| P2 |  |  | <0.01 | <0.001 |  |  | <0.01 | <0.001 |
| P3 |  |  |  | 0.053 |  |  |  | 0.065 |
| Flexion |  |  |  |  |  |  |  |  |
| P0 | <0.001 | <0.001 | <0.001 | <0.001 | <0.001 | <0.001 | <0.001 | <0.001 |
| P1 |  | <0.001 | <0.001 | <0.001 |  | <0.001 | <0.001 | <0.001 |
| P2 |  |  | 0.076 | <0.001 |  |  | 0.061 | <0.001 |
| P3 |  |  |  | <0.001 |  |  |  | <0.001 |
| Valgus |  |  |  |  |  |  |  |  |
| P0 | <0.001 | <0.001 | <0.001 | <0.001 | <0.001 | <0.001 | <0.001 | <0.001 |
| P1 |  | <0.001 | <0.001 | <0.001 |  | <0.001 | <0.001 | <0.001 |
| P2 |  |  | <0.01 | <0.001 |  |  | <0.01 | <0.001 |
| P3 |  |  |  | <0.01 |  |  |  | <0.01 |
| Varus |  |  |  |  |  |  |  |  |
| P0 | <0.001 | <0.001 | <0.001 | <0.001 | <0.001 | <0.001 | <0.001 | <0.001 |
| P1 |  | <0.001 | <0.001 | <0.001 |  | <0.001 | <0.001 | <0.001 |
| P2 |  |  | <0.05 | <0.01 |  |  | <0.05 | <0.01 |
| P3 |  |  |  | <0.05 |  |  |  | <0.05 |
