# Supplementary material for: Biomechanical comparison of screw-based zoning of PHILOS and Fx proximal humerus plates
Source: BMC Musculoskelet Disord. 2018 Jul 25;19:253. doi: 10.1186/s12891-018-2185-5 (PMC6060456; doi:10.1186/s12891-018-2185-5)
Supplement: Supplementary file 4 — Results of pairwise comparison statistical analysis: P values for plastic load at 15 mm displacement before (F15a) and after (F15b) eight-minute intermission and at 30 mm displacement (F30), for PHILOS plate configuration groups. (DOCX 15 kb) [file 12891_2018_2185_MOESM4_ESM.docx]

| Direction/Zone | P1 | P2 | P3 | P4 |
| --- | --- | --- | --- | --- |
| F_15a_ (N) |  |  |  |  |
| P0 | <0.001 | <0.001 | <0.001 | <0.001 |
| P1 |  | <0.001 | <0.001 | <0.001 |
| P2 |  |  | <0.001 | <0.001 |
| P3 |  |  |  | <0.05 |
| F_15b_ (N) |  |  |  |  |
| P0 | <0.001 | <0.001 | <0.001 | <0.001 |
| P1 |  | <0.001 | <0.001 | <0.001 |
| P2 |  |  | <0.001 | <0.001 |
| P3 |  |  |  | <0.05 |
| F_30_ (N) |  |  |  |  |
| P0 | <0.001 | <0.001 | <0.001 | <0.001 |
| P1 |  | <0.001 | <0.001 | <0.001 |
| P2 |  |  | <0.001 | <0.001 |
| P3 |  |  |  | <0.01 |
